# Cellular Traction Force Holds the Potential as a Drug Testing Readout for In Vitro Cancer Metastasis

**DOI:** 10.1007/s12195-024-00811-4

**Published:** 2024-07-04

**Authors:** Hui Yan Liew, Xiao Hui Liew, Wei Xuan Lin, Yee Zhen Lee, Yong Sze Ong, Satoshi Ogawa, Lor Huai Chong

**Affiliations:** 1 School of Pharmacy, Monash University Malaysia, Bandar Sunway, 47500 Subang Jaya, Malaysia; 2 Jeffrey Cheah School of Medicine and Health Sciences, Monash University Malaysia, Bandar Sunway, 47500 Subang Jaya, Malaysia

**Keywords:** Breast cancer, Metastasis, Cellular traction force, MCF-7, MDA-MB-231, Drug testing readout

## Abstract

**Introduction:**

Metastasis is responsible for 90% of cancer-related deaths worldwide. However, the potential inhibitory effects of metastasis by various anticancer drugs have been left largely unexplored. Existing preclinical models primarily focus on antiproliferative agents on the primary tumor to halt the cancer growth but not in metastasis. Unlike primary tumors, metastasis requires cancer cells to exert sufficient cellular traction force through the actomyosin machinery to migrate away from the primary tumor site. Therefore, we seek to explore the potential of cellular traction force as a novel readout for screening drugs that target cancer metastasis.

**Methods:**

In vitro models of invasive and non-invasive breast cancer were first established using MDA-MB-231 and MCF-7 cell lines, respectively. Cellular morphology was characterized, revealing spindle-like morphology in MDA-MB-231 and spherical morphology in MCF-7 cells. The baseline cellular traction force was quantified using the Traction force Microscopy technique. Cisplatin, a paradigm antimetastatic drug, and 5-Fluorouracil (5FU), a non-antimetastatic drug, were selected to evaluate the potential of cellular traction force as a drug testing readout for the in vitro cancer metastasis.

**Results:**

MDA-MB-231 cells exhibited significantly higher baseline cellular traction force compared to MCF-7 cells. Treatment with Cisplatin, an antimetastatic drug, and 5-Fluorouracil (5FU), a non-antimetastatic drug, demonstrated distinct effects on cellular traction force in MDA-MB-231 but not in MCF-7 cells. These findings correlate with the invasive potential observed in the two models.

**Conclusion:**

Cellular traction force emerges as a promising metric for evaluating drug efficacy in inhibiting cancer metastasis using in vitro models. This approach could enhance the screening and development of novel anti-metastatic therapies, addressing a critical gap in current anticancer drug research.

## Introduction

Cancer remains a leading cause of death worldwide, with over 90% of cancer-related deaths attributed to metastasis [1]. Metastasis occurs when transformed cells spread from their original site to distant locations, leading to the growth of secondary tumor colonies [1–3]. Acquiring an invasive phenotype distinguishes malignant from benign tumors, making it a crucial cancer characteristic [1–3].

However, current preclinical studies mainly focus on antiproliferative agents to halt primary tumor growth but overlook their impact on metastasis [4]. Similarly, clinical drug development often prioritizes triggering tumor shrinkage without considering the ability to inhibit metastasis [4]. Nevertheless, tumor shrinkage does not always lead to improved patient survival due to metastasis occurrence [5]. For example, around one-third of early-stage breast cancer patients have been shown to develop metastatic breast cancer [6, 7]. Therefore, there is an unmet need for in vitro drug screening models targeting metastatic processes, including cancer cell migration and invasion, rather than primarily focusing on tumor growth.

The Boyden chamber assay is a commonly employed in vitro method for investigating cell migration and invasion [8]. However, it has certain limitations when it comes to exploring the cellular pathways and interactions involved in cancer metastasis. Furthermore, the real-time imaging of migrating cells is not feasible due to the upside-down setup of the system. Therefore, we postulate that cellular traction force, which can recapitulate tumor-matrix interactions and reflect the downstream effector mechanisms of cell invasion, while also enabling the measurement of cell contractility, could serve as a promising readout for drug screening in the context of cancer metastasis.

Cellular traction forces are forces exerted by cells on their environment controlled by actomyosin [9], which serve as a useful readout to examine actomyosin contractility and cell migration. The cellular actomyosin machinery generates internal tension, leading to the contraction of the cell body to exert cellular traction forces on the underlying substrate. These forces are transmitted through focal adhesions, which serve as connections between the actin cytoskeleton and the extracellular matrix (ECM) [10]. Therefore, examining the cell contractility are critical as they are essential in all invasion and/or three-dimensional (3D) migration mechanisms, regardless of their protease dependence [11]. Multiple studies have explored the potential of cell traction forces to assess various cell properties, including focal adhesion dynamics, stress propagation, cell differentiation, and invasiveness [12–14]. Particularly, cellular traction force has emerged as a biomarker of metastatic potential and malignancy. Metastatic cells, such as those in breast, prostate, and lung cancer models, have been found to exert higher traction forces compared to non-metastatic cells [12, 15, 16]. However, the use of cellular traction force to assess the metastasis effect in different anticancer drugs remains largely unexplored.

In this study, we aim to investigate the potential of cellular traction force as a novel readout for drug screening targeting cancer metastasis. To begin, we first established the cellular traction force readout to assess the metastasis potential of both invasive and non-invasive cancer models, using MDA-MB-231 and MCF-7 breast carcinoma cells, respectively. We then compared the cellular traction force with the respective morphology and migration assays. We also observed how cell morphology changed after treating the cells with known antimetastatic drug Cisplatin and non-antimetastatic drug 5-Fluorouracil (5FU), at various concentrations within their IC_50_ range. Subsequently, we measured cellular traction force generated by MDA-MB-231 and MCF-7 to evaluate the antimetastatic potential of these anti-cancer drugs, to distinguish between the known antimetastatic drug, Cisplatin, from the non-antimetastatic drug, 5FU. Lastly, to further validate cellular traction force as a potential marker for anti-metastatic drug screening, we compared our findings with the cell invasion results from the Boyden chamber, the conventional cell invasion assay.

## Materials and Methods

### Materials

All drugs, chemicals, and reagents were purchased from Sigma Aldrich, USA, unless otherwise specified.

### Cell Culture

The human breast carcinoma cell lines MDA-MB-231 (RRID:CVCL_0062) and MCF-7 (RRID:CVCL_0031) were the gift from Dr. Yong Sze Ong’s lab from Monash University Malaysia. The cells were cultured in Dulbecco’s Modified Eagle Medium (DMEM) (Corning, USA) supplemented with 10% Fetal Bovine Serum (FBS) (Invitrogen, USA) and 1% penicillin-streptomycin (Invitrogen, USA). All cells were grown and maintained in a humidified incubator at 37 °C with 5% carbon dioxide. Cells were passaged at approximately 70-80% confluency prior to use.

### Drug Testing

The anticancer drugs utilized in this study were Cisplatin and 5FU. These drugs were initially dissolved as stock solutions in 100% dimethylsulfoxide (DMSO). Subsequently, working solutions were prepared by dissolving the stock solution in DMEM to ensure that the final concentration of DMSO within the medium did not exceed 0.1%. The specific drug concentrations employed in this study were determined based on the solubility of the drugs and the associated IC_50_ values for each drug component. Prior to commencing drug testing, MDA-MB-231 and MCF-7 cells were seeded at a density of 2 × 10^4^ cells on 12 mm glass-covered slips within 24-well plates. These cells were then incubated for 24 h to ensure proper cell attachment. After 24 h, MDA-MB-231 cells were exposed to varying concentrations of each drug compound: Cisplatin at 20, 40, and 60 µM, and 5FU at 30, 40, and 50 µM. Meanwhile, MCF-7 cells were subjected to different concentrations of each drug compound: 2.5, 10, and 12.5 µM for Cisplatin, and 5FU at 20, 30, and 40 µM. Following the 24-h incubation period, the cells were stained with Alexa Fluor 488-conjugated Phalloidin (Invitrogen, USA, diluted 1:500) and DAPI (Invitrogen, USA, diluted 1:1000) to examine the cell morphology, as described in the immunostaining section.

### Immunostaining

A cell density of 2 × 10^4^ cells was seeded on 12 mm glass-covered slips in 24-well plates and incubated for 24 h before performing immunostaining to examine the cell morphology of invasive and non-invasive cancer cells. After the cell seeding and the drug testing as mentioned previously, the cells were fixed with 3.7% paraformaldehyde for 20 min at room temperature and permeabilized with 0.1% Triton-X for 15 min at room temperature. Subsequently, the cells were blocked with 2% bovine serum albumin for 1 h. F-Actin fibers were stained with Alexa Fluor 488-conjugated phalloidin (Invitrogen, USA, diluted 1:500) for 1 h at room temperature, while the nuclei were counterstained with DAPI (Invitrogen, USA, diluted 1:1000). The fixed samples were imaged using a Ti2 Nikon fluorescence microscope with an ×40 objective lens.

### Image Analysis for Cell Morphology

To analyze cell morphology, immunofluorescence images of F-actin were used. These fluorescent images were processed and analyzed using *Image J* software (RRID:SCR_003070). The quantification of cell morphological parameters, such as cell area, perimeter, circularity, and cell aspect ratio, was first processed by segmenting the F-actin using a threshold value determined through the *Otsu* method. A binary operation to fill holes was then applied to obtain the segmented cell area. Subsequently, the *Analyzing particles* function was used to determine the values of cell area, perimeter, and circularity (defined as 4 × *π* × area/(perimeter)^2^). The cell aspect ratio was calculated by dividing the length of its major axis by that of its minor axis.

### Migration and Invasion Assay

**Fig. 1:**
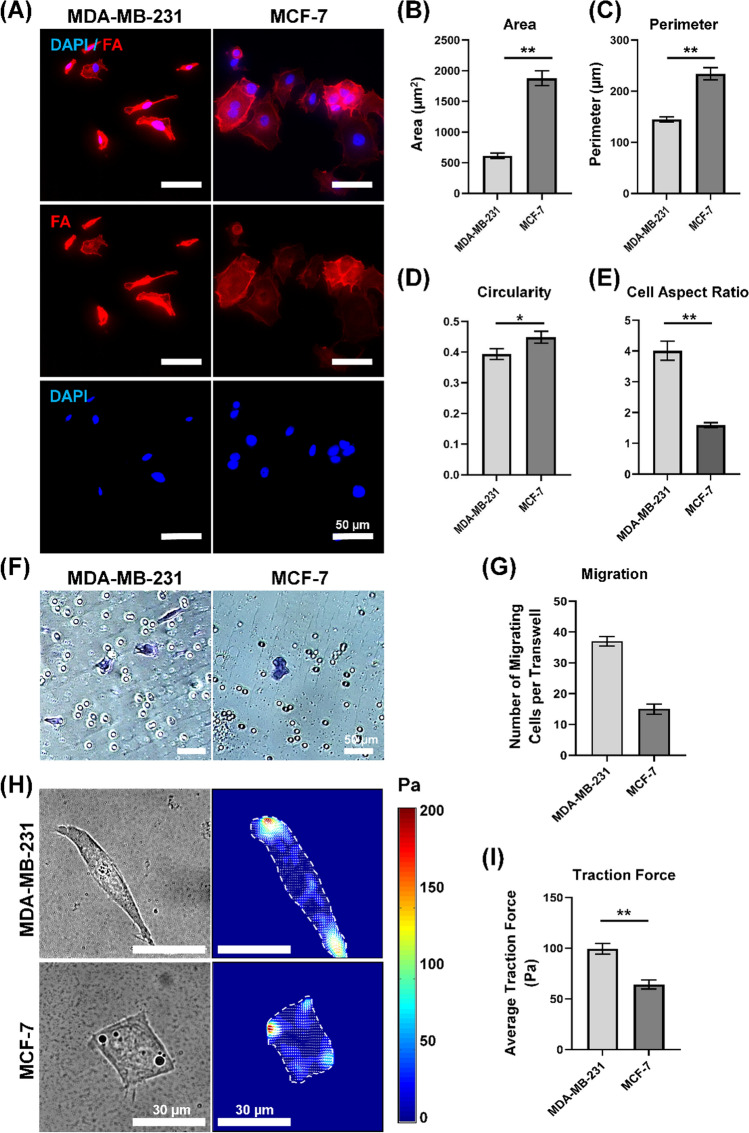
Cellular traction force increases in MDA-MB-231 driven by cellular morphology and migratory phenotype. **A** Immunofluorescence images of MDA-MB-231 and MCF-7 stained with DAPI (nuclei) (blue) and Phalloidin (F-actin) (red). Scale bar = 50 μm. Quantification of **B** Area, **C** Perimeter, **D** Circularity, **E** Cell aspect ratio of MDA-MB-231 and MCF-7. **F** Transwell migration assay of MDA-MB-231 and MCF-7 cells after 24 h. Scale bar = 50 μm. The image taken from the lower compartment of the Transwell shows migrating cells stained blue with a 0.5% crystal violet solution for visualization and quantification. **G** Quantification of the Transwell migration assay for MDA-MB-231 and MCF-7 cells. Both rounded and elongated cells are counted. Five random visual fields are selected from each insert to calculate the total number of migrating cells per transwell. **H** Brightfield images and Traction force heatmap of MDA-MB-231 and MCF-7. Scale bar = 30 μm **I** Average traction force magnitudes of MDA-MB-231 and MCF-7, *n* = 22 and *n* = 19 respectively. All the light grey bars denote the quantification of invasive model, MDA-MB-231 while the dark grey bar denote the quantification of non-invasive model, MCF-7. Data are the mean ± SEM of three independent experiments. Asterisks indicate statistically significant differences (Student *t* test, *p < 0.05, **p < 0.01)

The cell migration assay was performed using 24-well plates with 8-μm pore-sized chamber inserts (Corning, USA) to assess the migratory behavior of cancer cells between invasive and non-invasive models in Transwell chambers with non-coated membranes (24-well insert, pore size: 8 mm, Corning, USA). Meanwhile, the cell invasion assay was carried out to specifically examine the impact of drug treatment on the invasive properties of MDA-MB-231 cells. In the context of invasion assay, the upper surface of a filter membrane in the upper compartment of a Transwell was coated with 30 µg of Matrigel overnight prior to usage. Approximately 1 × 10^5^ cells/well of MDA-MB-231 (both in the migration and invasion assay) and MCF-7 (only in the migration assay) were resuspended in 200 μL of serum-free medium and seeded into the upper chamber of each insert with the non-coated membrane for migration assay and Matrigel-coated membrane for invasion assay. The cells were incubated overnight to allow cell attachment. Then, 500 μL of DMEM containing 10% FBS was added to the lower chamber of a 24-well plate. For the invasion assay, drug treatment was conducted after 24 h of cell attachment. The cells were then exposed to the drug treatment for an additional 24 h prior to the fixation and staining process. After that, the migrated or invaded cells were first washed twice with PBS and then fixed using a 4% paraformaldehyde solution. Subsequently, they were stained for 15 min with a 0.5% crystal violet solution. Any cells that had not migrated or invaded the lower compartment were carefully removed with a cotton swab. After staining, the migrating or invading cells appeared blue due to the crystal violet staining, and phase contrast images were taken. To quantify the migrating cells in Fig. 1G, five random visual fields were selected from each insert to calculate the total number of migrating cells per transwell. The number of migrating cells per transwell is calculated as follows:

$${\text{Number}}\;{\text{of}}\;{\text{migrating}}\;{\text{cells}}\;{\text{per}}\;{\text{transwell}} = {\text{Total}}\;{\text{number}}\;{\text{of}}\;{\text{migrating}}\;{\text{cells}}\;{\text{from}}\;{\text{five}}\;{\text{random}}\;{\text{visual}}\;{\text{fields}}\;{\text{per}}\;{\text{each}}\;{\text{transwell}}\;{\text{insert}}$$

The average number of migrating cells was calculated based on three independent experiments, each conducted in triplicate.

To quantify cell invasion for Fig. 5F, five random visual fields were selected from each insert to calculate the number of invading cells per transwell. Following image analysis and quantification, the percentage of invaded cells treated with cisplatin and 5FU was determined. This was done by normalizing the count of drug-treated cells to that of their untreated vehicle control (MDA-MB-231 cells in 0.1% DMSO), using the following formula:

$${\text{Invaded}}\;{\text{cells}}\;\left( \% \right) = \frac{{{\text{Number}}\;{\text{of}}\;{\text{migrating}}\;{\text{cells}}\;{\text{after}}\;{\text{drug}}\;{\text{treatment}}}}{{{\text{Number}}\;{\text{of}}\;{\text{migrating}}\;{\text{cells }}\;{\text{in}}\;{\text{the}}\;{\text{vehicle}}\;{\text{control}} \left( {0.1\% {\text{DMSO}}} \right)}} \times 100\%$$

However, MCF-7 cells were not assessed for invasion in Fig. 5 as they did not readily invade through the Matrigel-coated inserts under our experimental conditions.

### Fabrication of Polyacrylamide (PAA) Gel Substrates

Prior to the preparation of the polyacrylamide (PAA) gel, glass coverslips with a diameter of 25 mm were initially treated with a silane solution comprised of 1.2% 3-methacryloxypropyltrimethoxysilane and 2% acetic acid, for 2 h at room temperature to improve the adhesion of the PAA gel to the glass substrate. The preparation of the PAA gels involved co-polymerizing bis-acrylamide (Bio-Rad, CA) with N-acryloyl-6-aminocaproic acid (ACA; Tokyo Chemical Industry, Japan) to enhance the stability of protein binding within the PAA gels. As a result, 100 mM of ACA was introduced into the 40% bis-acrylamide gel solution to yield 14 kPa of ACA-co-polymerized gels on the silanized glass coverslips. For the purpose of visualizing substrate deformation and calculating the magnitude of traction force exerted by the cells, green fluorescent beads with a diameter of 0.2 μm (Life Technologies, USA) were integrated into the gel solution. PAA gel polymerization was initiated by adding ammonium persulfate (APS) and tetramethylethylenediamine (TEMED) (Bio-Rad, Hercules, CA) to the gel solution, each at a final concentration of 0.1%. A 4 μL gel solution was placed on the silanized coverslip and then covered with a 12 mm circular coverslip that had not been treated. This sandwiched configuration was incubated for 10 min at 37 °C. Upon gel polymerization after 10 min, the circular coverslip was gently removed. Subsequently, the polymerized PAA gels were fully hydrated in MES buffer [0.1 M 2-(N-morpholino) ethanesulfonic acid, 0.5 M sodium chloride, pH 6.1]. To enhance cell attachment to the PAA surface, the gels were pre-coated with 50 µg/mL fibronectin, an extracellular matrix protein. To immobilize fibronectin on the surface of ACA-co-polymerized gels, a dehydration condensation reaction was carried out using a water-soluble carbodiimide by activating the carboxyl groups of the ACA gels using a solution of 0.5 M N-hydroxysuccinimide (NHS, Wako Pure Chemical Industries, Osaka, Japan) and 0.2 M 1-ethyl-3-(3-dimethylaminopropyl) carbodiimide hydrochloride (EDAC, Dojindo Laboratories, Kumamoto, Japan) for 30 min at room temperature on a shaker. Following the activation, the gels were washed with 60% cold methanol diluted with PBS for 1 h at 4 °C. Subsequently, the surfaces of the ACA-co-polymerized gels were conjugated with 50 µg/mL fibronectin (Roche, USA) at 4 °C overnight on a shaker. Finally, the gels were transferred to a solution of 0.5 M ethanolamine diluted with HEPES buffer [0.5 M HEPES, pH 9.0] for 30 min at 4 °C. Following these steps, the gels were subjected to two washes with HEPES buffer at 4 °C, followed by three washes using PBS. Before cell seeding, the gels were sterilized with UV light for 15 min.

### Traction Force Analysis

To assess the potential of cellular traction force as a readout for antimetastatic drug testing, 1 × 10^4^ cells/well were seeded onto 14 kPa PAA gels attached to glass coverslips, following previously published protocols [17]. In brief, the gels were first functionalized with 50 μg/mL fibronectin diluted in HEPES buffer (0.5 M HEPES, pH9.0). Subsequently, cells were seeded onto the fibronectin-coated PAA gels and allowed to attach for a 24-h period. Live-cell imaging was then performed. For the purpose of drug testing, subsequent to the initial 24-h interval of cell attachment, the cells were exposed to 40 µM of Cisplatin and 40 µM of 5FU for an additional 24-h period. The live-cell imaging was conducted using the AF6000 Leica LIVE inverted fluorescent microscope equipped with a × 60 oil objective lens (Numerical Aperture 1.4). The details of the two-dimensional (2D) traction force calculations were described in Yip’s work [17]. In brief, two sets of images of the fluorescent beads on the gel surface were captured before and after cell detachment using 10% trypsin. The 2D displacement vectors, arising due to the deformation of the gel resulting from cell-induced traction forces, were determined through the application of the digital image correlation algorithm, as developed by Franck et al. [18, 19]. After acquiring the displacement vectors, the stress tensor ε of the gel was calculated using the displacement gradient technique. Subsequently, the material stress tensor σ could then be computed based on the material’s constitutive relation, given by *σ* = *Eε*/(1 + *v*) where E represents Young's modulus of the gel, and v stands for the Poisson's ratio of the gel (with *v* = 0.5).

### Cell Viability Assay

MDA-MB-231 cells at a density of 1 × 10^4^ were seeded onto 96-well plates and allowed to incubate for 24 h to ensure cell attachment prior to drug testing. Subsequently, the cells were exposed to various concentrations of Cisplatin and 5FU (0 µM, 1.95 µM, 3.91 µM, 7.81 µM, 15.63 µM, 31.25 µM, 62.5 µM, 125 µM, 250 µM, and 500 µM) for an additional 24 h. Following this incubation period, the cells were treated with MTT (3-(4,5-dimethylthiazol-2-yl)− 2,5-diphenyltetrazolium bromide) solution (0.5 mg/mL) for 3 h (Elabsciences), and the resulting formazan precipitate was dissolved in 150 µL of DMSO. The absorbance measurements of each well were then measured at 570 nm using a microplate spectrophotometer (SpectraMax iD3). Each experiment was conducted independently in triplicate. The results of the Cisplatin and 5FU treatments on MDA-MB-231 were analyzed, and dose-response curves as well as IC_50_ values were calculated using GraphPad Prism 9.0 (USA, RRID:SCR_002798)

### Statistical Analysis

All data were presented as the mean value ± standard error of the mean (SEM). Student's *t* test was used to analyze the statistical significance of the data between two groups using GraphPad Prism 9.0 (USA, RRID:SCR_002798). The means of the two datasets were significantly different if the following p-values were obtained: *p* < 0.05 (*), *p* < 0.01 (**).

## Results

### Cellular Traction Force Increases in Metastatic Cancer Model Driven by Cellular Morphology and Migratory Phenotype

To investigate the relationship between cellular traction force generation and metastatic potential, we examined the differences in magnitude of traction force generated by metastatic and non-metastatic cancer models. As cancer cells progress through metastasis, they undergo phenotypic changes that alter their adhesion and migration behaviors, enabling the cancer cells to detach from the primary tumor and infiltrate surrounding tissue. We postulate that metastatic cancer cells, in order to metastasize, require increased contractile force generation compared to non-metastatic cells. Given that invasion marks the initial phase of the metastatic process, we first selected the invasive breast cancer cell line, MDA-MB-231, and the non-invasive breast cancer cell line, MCF-7, to serve as representatives of the metastatic and non-metastatic states, respectively. We then conduct a comprehensive analysis of cell morphology, migration assays, and cellular traction force measurements on both MDA-MB-231 and MCF-7 cells.

In Fig. 1A, we observed that MDA-MB-231 cells exhibited a spindle-like shape, while MCF-7 cells displayed an epithelial-like morphology from the fluorescent images. Our quantitative analysis in Fig. 1B supported this observation by demonstrating that the area of the MDA-MB-231 (597.78 ± 37.85 µm^2^) was significantly smaller than that of the MCF-7 (1878.74 ± 119.76 µm^2^). This trend persists for perimeter measurements, as indicated in Fig. 1C. MDA-MB-231 cells exhibited a significantly lower perimeter (144.91 ± 4.92 µm) in contrast to MCF-7 cells (234.21 ± 11.86 µm). In Fig. 1D, MCF-7 cells showed a more circular morphology with a higher circular index of 0.45 ± 0.019 and a lower cell aspect ratio of 1.59 ± 0.08. In contrast, MDA-MB-231 cells displayed a less circular shape with elongated and narrow morphology, as evidenced by their lower circular index of 0.39 ± 0.017 and a high cell aspect ratio of 4.0 ± 0.31 (Fig. 1E). Overall, MDA-MB-231 cells demonstrated a significantly smaller and elongated morphology than MCF-7 cells (Fig. 1E). We then conducted a migration assay using an 8 µm pore size transwell system, detailed in Fig. 1F and G. In this assay, a suspension of cells in serum-free DMEM was added to the upper chamber of each insert with a non-coated membrane, while 500 µL of DMEM containing 10% FBS was introduced into the lower chamber to serve as a chemoattractant. After 24 h of incubation, cells that had migrated to the lower compartment were fixed and stained with 0.5% of crystal violet solution for visualization and quantification.

In Fig. 1F, we observed that MCF-7 demonstrated a more circular morphology. These cells generally exhibit a non-invasive and circular morphology. In contrast, the rounded cells observed in MDA-MB-231 samples are likely due to the physical constraints imposed by the 8 μm pore size of the transwell used in the invasion assay. Given that the diameter of both MCF-7 and MDA-MB-231 cells exceeds 10 μm, this confinement necessitates a rounded morphology for the cells to navigate through the narrow pores of the transwell, facilitating their migration in such restricted environments. Cells often temporarily round up as a response to the mechanical constraints imposed by the small pores of the transwell apparatus, minimizing contact area with the pore walls to ease passage through confined spaces. After navigating these constraints, cells may remain rounded due to slow cytoskeletal reorganization returning to pre-stress conditions. This phenomenon is well-documented in cell migration research and our result is consistent with previously reported findings [20–22], which noted the presence of rounded MDA-MB-231 cells in the lower compartment of an 8 μm-pore-sized transwell post-invasion.

To accurately quantify migration, we selected five random visual fields from each insert. We counted the total number of migrating cells per transwell, noting both circular and elongated morphologies, and presented these findings in Fig. 1F. Further details about the quantification process for the migrating cells shown in Fig. 1F can be found in "Migration and Invasion Assay" section of the methodology.

Our findings indicated that MDA-MB-231 cells demonstrated an increased migratory capacity (Fig. 1G), exhibiting a greater number of migrated cells (37 ± 1.57), in comparison to MCF-7 cells (15 ± 1.64). Lastly, we conducted traction force microscopy to measure cellular traction force in Fig. 1H and I. Our results demonstrated that MDA-MB-231, a highly metastatic and invasive breast cancer cell, generated a significantly higher average traction force (99.4 ± 5.2 kPa) as compared to the non-metastatic/non-invasive MCF7 cells (64.2 ± 4.5 kPa).

Taking all these into consideration, MDA-MB-231, as the metastatic and invasive cell model, exhibited a more elongated structure with enhanced migratory activity and a higher traction force compared to the non-metastatic and non-invasive cell model, MCF-7. These findings suggest that increasing traction force generation in invasive cell models demonstrating high metastatic potential, could potentially serve as a valuable biophysical marker for metastatic cells.

### MDA-MB-231 Demonstrates a Significantly Smaller and more Circular Morphology in Response to Cisplatin but Not in 5FU

Prior to investigating the potential use of cellular traction force as a drug testing readout for in vitro cancer metastasis models, we have selected Cisplatin and 5FU as our paradigm anti-metastatic drug and non-antimetastatic drugs, respectively. Cisplatin was chosen due to its antimetastatic properties to impede the Epithelial-Mesenchymal Transition (EMT), thereby inhibiting cell migration, invasion, and ultimately, metastasis. On the other hand, 5FU is selected as our non-antimetastatic drug option due to its ability to inhibit tumor growth and proliferation without impacting invasion, making it an ideal point of comparison.

**Fig. 2:**
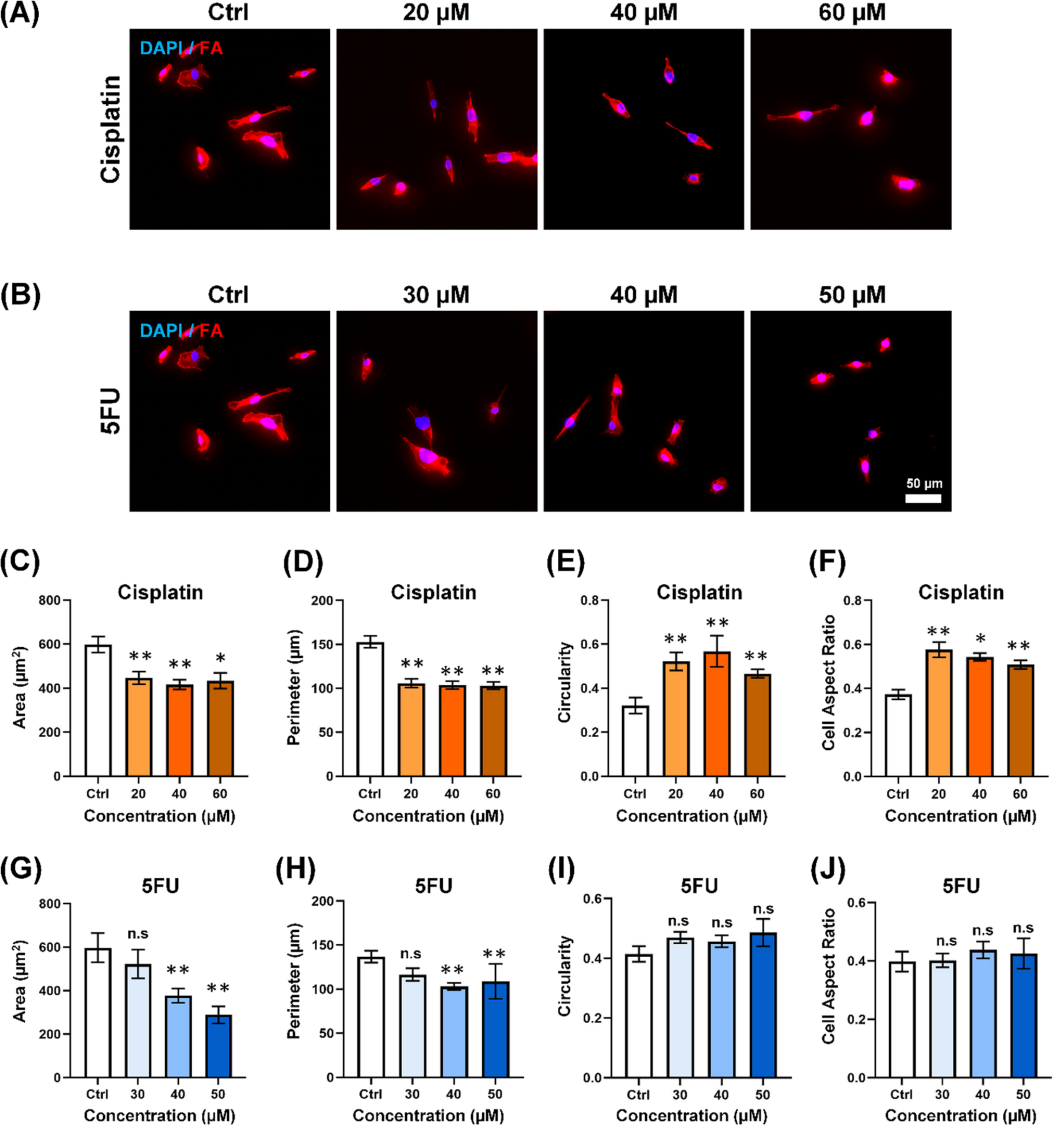
MDA-MB-231 demonstrates a significantly smaller and more circular morphology in response to Cisplatin but not in 5FU. Immunofluorescence images of MDA-MB-231 and MCF-7 stained with the DAPI (nuclei) (blue) and Phalloidin (F-actin) (red) after being treated with **A** Cisplatin and **B** 5FU. Scale bar = 50 μm. Quantification of **C** Area, **D** Perimeter, **E** Circularity, **F** Cell aspect ratio of MDA-MB-231 after being treated with Cisplatin. The white bars denote the control group while the light brown to the dark brown bar denotes the increasing concentration of Cisplatin from 20 uM to 40 uM to 60 uM. Quantification of **G** Area, **H** Perimeter, **I** Circularity, **J** Cell aspect ratio of MDA-MB-231 after being treated with 5FU. The white bars denote the control group while the light blue to the dark blue bar denotes the increasing concentration of 5FU from 30 uM to 40 uM to 50 uM. Data are the mean ± SEM of three independent experiments. Asterisks indicate statistically significant differences (Student *t* test, *p < 0.05, **p < 0.01) while n.s indicates statistically non-significant differences.

We first assessed the effects of both the antimetastatic drug Cisplatin and the non-antimetastatic drug 5FU on the cellular morphology of MDA-MB-231 cells, the invasive breast cancer model. Our findings in Fig. 2A and B demonstrated a reduction in cell size upon exposure to varying concentrations of Cisplatin and 5FU. We further found that the Cisplatin-treated MDA-MB-231 cells demonstrated a significant decrease in area, from 598.32 ± 36.67 μm^2^ to 434.26 ± 35.60 μm^2^, as illustrated in Fig. 2C. Similarly, a reduction in perimeter from 152.96 ± 6.67 μm to 103.12 ± 4.27 μm was shown in Fig. 2D, with increasing Cisplatin concentration up to 60 μM. In terms of circularity, the elongated MDA-MB-231 exhibited a more circular morphology after being treated with Cisplatin, as reflected by the circularity index shifting from 0.37 ± 0.022 to high circularity 0.52 ± 0.016 in the 60 μM Cisplatin-treatment group (Fig. 2E). Similarly, the cell aspect ratio decreased from 4.42 ± 0.44 (control) to 3.06 ± 0.21 after exposure to 60 μM Cisplatin (Fig. 2F). Given that a higher cell aspect ratio refers to a more elongated structure, while a lower cell aspect ratio indicates a more circular morphology, the antimetastatic drug, Cisplatin triggered a shift towards a more circular morphology on MDA-MB-231. However, the observations were different when MDA-MB-231 cells were subjected to 5FU treatment.

In Fig. 2G and H, a significant reduction was observed in both the area and perimeter of MDA-MB-231 cells upon treatment with 5FU at concentrations of 40 μM and 50 μM. Specifically, the area of MDA-MB-231 cells decreased from 597.25 ± 66.85 μm^2^ (control) to 377.13 ± 32.86 μm^2^ and 288.74 ± 39.07 μm^2^ with treatments of 40 μM and 50 μM of 5FU, respectively. The perimeter of MDA-MB-231 cells in the 5FU treatment groups also decreased from 136.87 ± 6.97 μm to 108.85 ± 20.96 μm upon exposure to 50 μM of 5FU. Interestingly, we found no significant increase in circularity between the control group and the 5FU-treated group, as evidenced by the circularity index shifting from 0.41 ± 0.027 to 0.49 ± 0.048 in Fig. 2I, and the cell aspect ratio changing from 3.6 ± 0.43 to 3.14 ± 0.43 after treatment with 50 μM 5FU (Fig. 2J).

While 5FU effectively decreased the size of MDA-MB-231 cells, it did not induce circular morphology in these cells. As a result, MDA-MB-231 cells maintained their elongated morphology even after the 5FU treatment. These interesting observations suggest that Cisplatin, an antimetastatic drug, not only reduces cell size but also triggers the circular morphology of MDA-MB-231 to inhibit the invasive nature of metastatic cells. In contrast, 5FU, is an anti-proliferative agent that targets cell growth, leaving invasive properties unaffected, resulting in a smaller yet elongated cellular morphology.

### MCF-7 Exhibits a Significantly Smaller and more Circular Morphology in Response to Cisplatin and 5FU

**Fig. 3:**
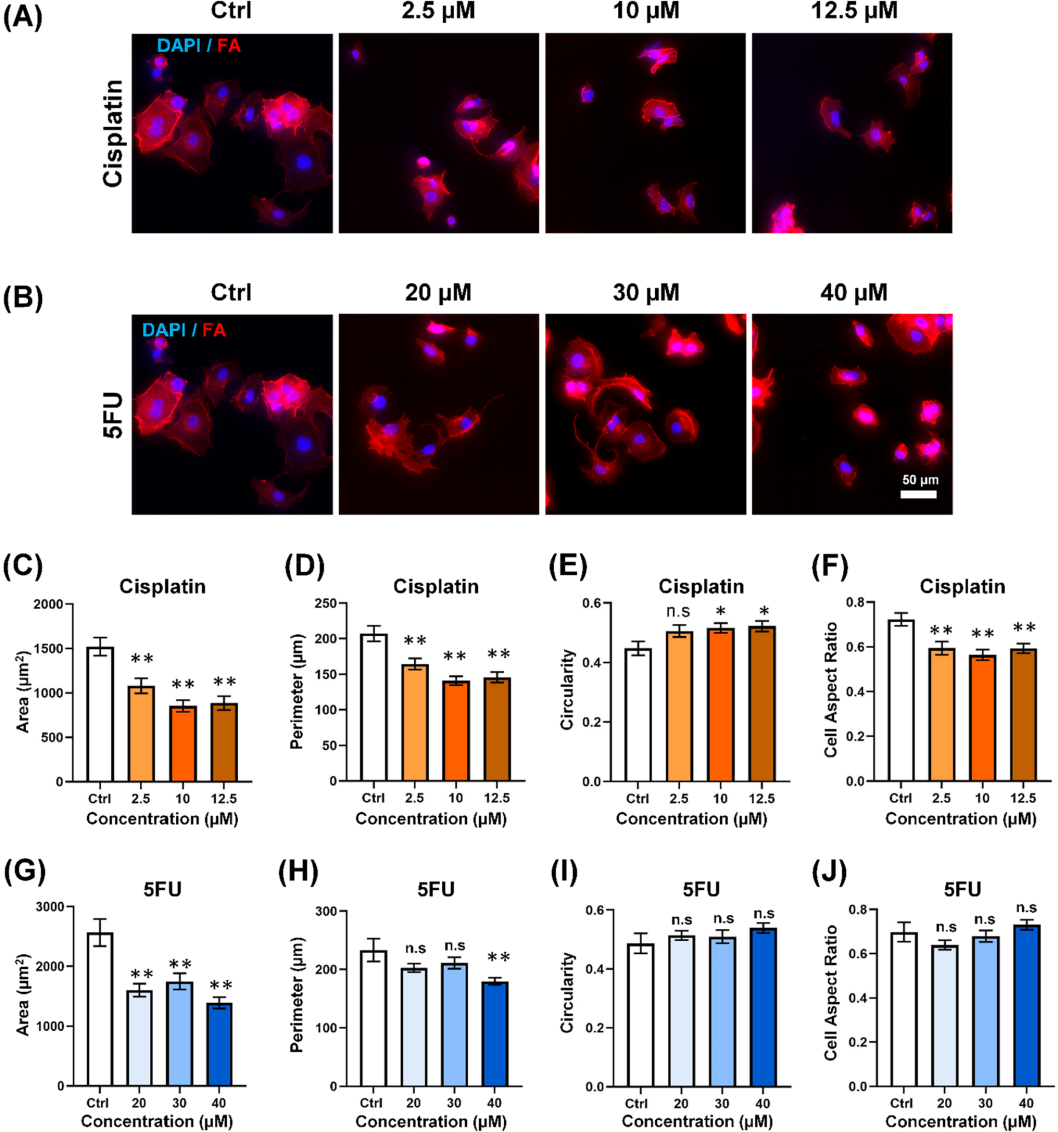
MCF-7 exhibits a significantly smaller and more circular morphology in response to Cisplatin and 5FU. Immunofluorescence images of MCF-7 stained with the DAPI (nuclei) (blue) and Phalloidin (F-actin) (red) after being treated with **A** Cisplatin and **B** 5FU. Scale bar = 50 μm. Quantification of **C** Area, **D** Perimeter, **E** Circularity, **F** Cell aspect ratio of MCF-7 after being treated with Cisplatin. The white bars denote the control group while the light brown to the dark brown bar denotes the increasing concentration of Cisplatin from 20 uM to 40 uM to 60 uM. Quantification of **G** Area, **H** Perimeter, **I** Circularity, **J** Cell aspect ratio of MCF-7 after being treated with 5FU. The white bars denote the control group while the light blue to the dark blue bar denotes the increasing concentration of 5FU from 30 uM to 40 uM to 50 uM. Data are the mean ± SEM of three independent experiments. Asterisks indicate statistically significant differences (Student *t* test, *p < 0.05, **p < 0.01) while n.s indicates statistically non-significant differences.

We then further investigate the effects of Cisplatin and 5FU on cell morphology in non-metastatic breast cancer model, MCF-7 in Fig. 3. Similarly, Fig. 3A and B demonstrated that the size of MCF-7 became smaller upon treatment with different concentrations of Cisplatin and 5FU. The quantification result in Fig. 3C and D demonstrated a decreasing trend in area, from 1520.80 ± 102.36 µm^2^ to 885.32 ± 77.77 µm^2^ in 12.5 μM Cisplatin, and perimeter, from 207.17 ± 10.86 µm to 145.77 ± 7.304 µm. For circularity morphology, Cisplatin triggers more circular morphology in MCF-7, from the circularity index of 0.45 ± 0.023 to 0.52 ± 0.018 after being treated by 12.5 μM, respectively, in Fig. 3E. However, the cell aspect ratio shown in Fig. 3F is higher, from 1.55 ± 0.10 to 2.0 ± 0.11.

For 5-FU, upon the quantification of the cell morphology, 5FU-treated MCF-7 cells demonstrated a significant decrease in area, from 2566.00 ± 237.30 µm^2^ to 1390.73 ± 96.69 µm^2^ shown in Fig. 3G. A similar trend was observed in the perimeter, from 286.13 ± 24.87 µm to 179.79 ± 6.16 µm, respectively, as shown in Fig. 3H. However, we observed that 5FU did not induce the circular morphology of the MCF-7, as the circularity index (Fig. 3I) and cell aspect ratio (Fig. 3J) did not demonstrate a significant change after being treated with 5FU.

As a summary, we found that Cisplatin induced a reduction in cell size and the circular morphology of MCF-7, whereas 5FU led to size reduction without substantial morphological changes. Given the epithelial-like nature of MCF-7, both Cisplatin and 5FU demonstrated a similar response, resulting in smaller and more circular morphology of non-metastatic cells.

### MDA-MB-231 Demonstrates Significant Differences in Cellular Traction Forces Magnitude Following Cisplatin and 5FU Treatments, Contrasting MCF-7 Responses

**Fig. 4:**
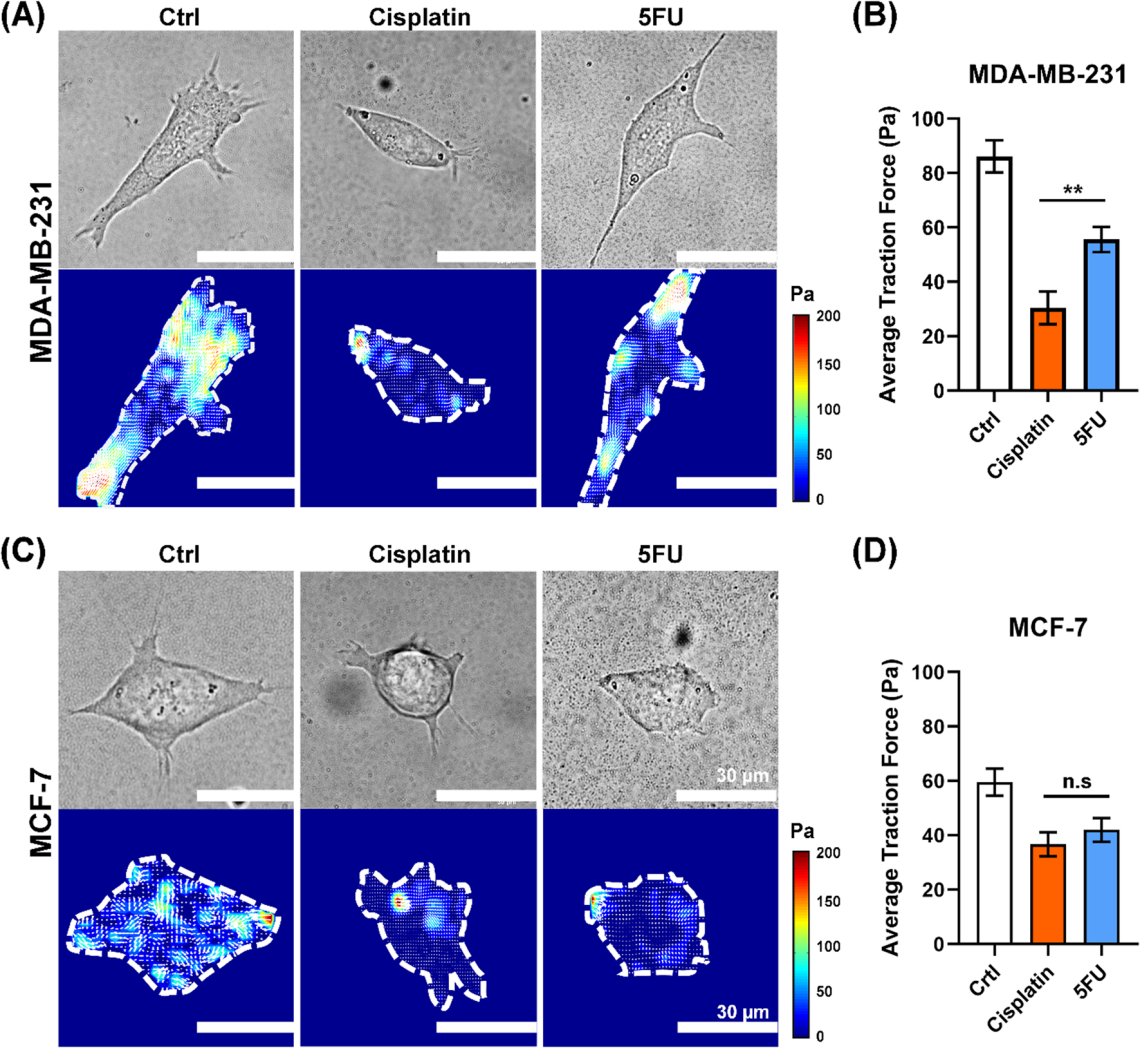
MDA-MB-231 demonstrates a significant differences of average cellular traction forces magnitude in response to Cisplatin and 5FU but not in MCF-7. **A** Brightfield images and Traction force map of MDA-MB-231 after 24-h drug treatment with 0.1% DMSO (Ctrl), 40 µM Cisplatin and 40 µM 5FU. **B** Average cellular traction force magnitudes of MDA-MB-231, *n* = 38. **C** Brightfield images and Traction force map of MCF-7 after 24-h drug treatment with 0.1% DMSO (Ctrl), 12.5 µM Cisplatin and 20 µM 5FU. **D** Average cellular traction force magnitudes of MCF-7, *n* = 32. All the white bars denote the quantification of the average traction force magnitude in the control condition (0.1% DMSO) while the brown bars denote the quantification of the average traction force magnitude after a 24-h Cisplatin treatment and blue bars denote the quantification of the average traction force magnitude after a 24-h of 5FU treatment. Data are the mean ± SEM. Asterisks indicate statistically significant differences (Student *t* test, *p < 0.05, **p < 0.01) while n.s indicates statistically non-significant differences. Scale bar: 30 μm

To assess the potential of cellular traction forces as a marker for in vitro drug testing in cancer metastasis, we measured the average cellular traction force magnitudes exhibited by MDA-MB-231 and MCF-7 cells following exposure to Cisplatin and 5FU, as depicted in Fig. 4. In order to precisely quantify the distribution of cellular traction forces within the single-cell level, we cultured these cells on PAA gels containing embedded fluorescent microbeads prior to drug testing.

In the context of drug testing, we first added Cisplatin and 5FU into the culture medium of MDA-MB-231 cells at final concentrations of 40 µM respectively, and subsequently assessed their effects on traction forces after a 24-h treatment period. Similarly, non-invasive MCF-7 cells were subjected to 12.5 µM Cisplatin and 20 µM 5FU. The selection of drug concentrations was based on the data obtained from Figs. 2 and 3.

Before administering the drugs, we found that MDA-MB-231 cells displayed an elongated structure (Fig. 4A), whereas MCF-7 cells exhibited a rounded, epithelial-like morphology (Fig. 4C) at the single-cell level, which aligned with our findings in Fig. 1.

Following 40 µM Cisplatin treatment, the elongated MDA-MB-231 cells experienced a morphological shift toward a more circular and rounded shape (Fig. 4A), resulting in a significant reduction in cellular traction force magnitude from 86.8 ± 5.9 kPa to 30.4 ± 6.1 kPa (Fig. 4B). In contrast, 5FU, primarily characterized as an anti-proliferation drug, induced a decrease in cell size without causing significant morphological changes (Fig. 4A). As a result, MDA-MB-231 cells retained their elongated shape but became smaller, leading to a slight decrease in average cellular traction force magnitude from 86.8 ± 5.9 kPa to 55.6 ± 4.62 kPa (Fig. 4B). Interestingly, when comparing the traction forces generated by MDA-MB-231 cells after drug treatments involving both anti-metastatic (Cisplatin) and non-anti-metastatic (5FU) drugs, we observed significant differences between these two drugs (Fig. 4B), highlighting the potential of cellular traction force to distinguish the anti-metastatic drug from the non-antimetastatic drug.

In contrast, following the exposure of 12.5 µM Cisplatin on MCF-7 cells, despite maintaining their circular and epithelial-like structure, there was a slight reduction in cell size (Fig. 4C), resulting in a lower cellular traction force magnitude of 36.7 ± 4.4 kPa (Fig. 4D). Similarly, treatment with 20 µM 5FU led to a decrease in cell size and a reduction in average cellular traction force magnitude (Fig. 4C) from 59.6 ± 4.9 kPa to 42 ± 4.8 kPa (Fig. 4D). It is worth noting that, we did not observe significant differences in the average cellular traction force generated by MCF-7 cells after treatment with anti-metastatic (Cisplatin) and non-anti-metastatic (5FU) drugs. This result suggested the specificity of cellular traction force as a biophysical marker targeting invasive cell models, which represent the initial step in the metastasis process.

Taken all these together, our findings demonstrated a significant decrease in average cellular traction forces generated by MDA-MB-231 cells when treated with Cisplatin compared to 5FU, whereas this effect was not observed in MCF-7 cells. These results indicate that cellular traction force may serve as a specific marker for invasive and metastatic models.

### MDA-MB-231 Invasion is Inhibited by Cisplatin but Not 5FU

Given that the invasion assay is the established gold standard for assessing cellular invasiveness and metastatic potential in cancer research, we conducted an invasion assay on MDA-MB-231 cells to cross-validate our cellular traction force findings and investigate the correlation between invasiveness and cellular traction force. It is also important to note that we excluded MCF-7 cells from this invasion study due to their non-invasive characteristics. As a result, this experiment was exclusively focused on MDA-MB-231 cells.

**Fig. 5:**
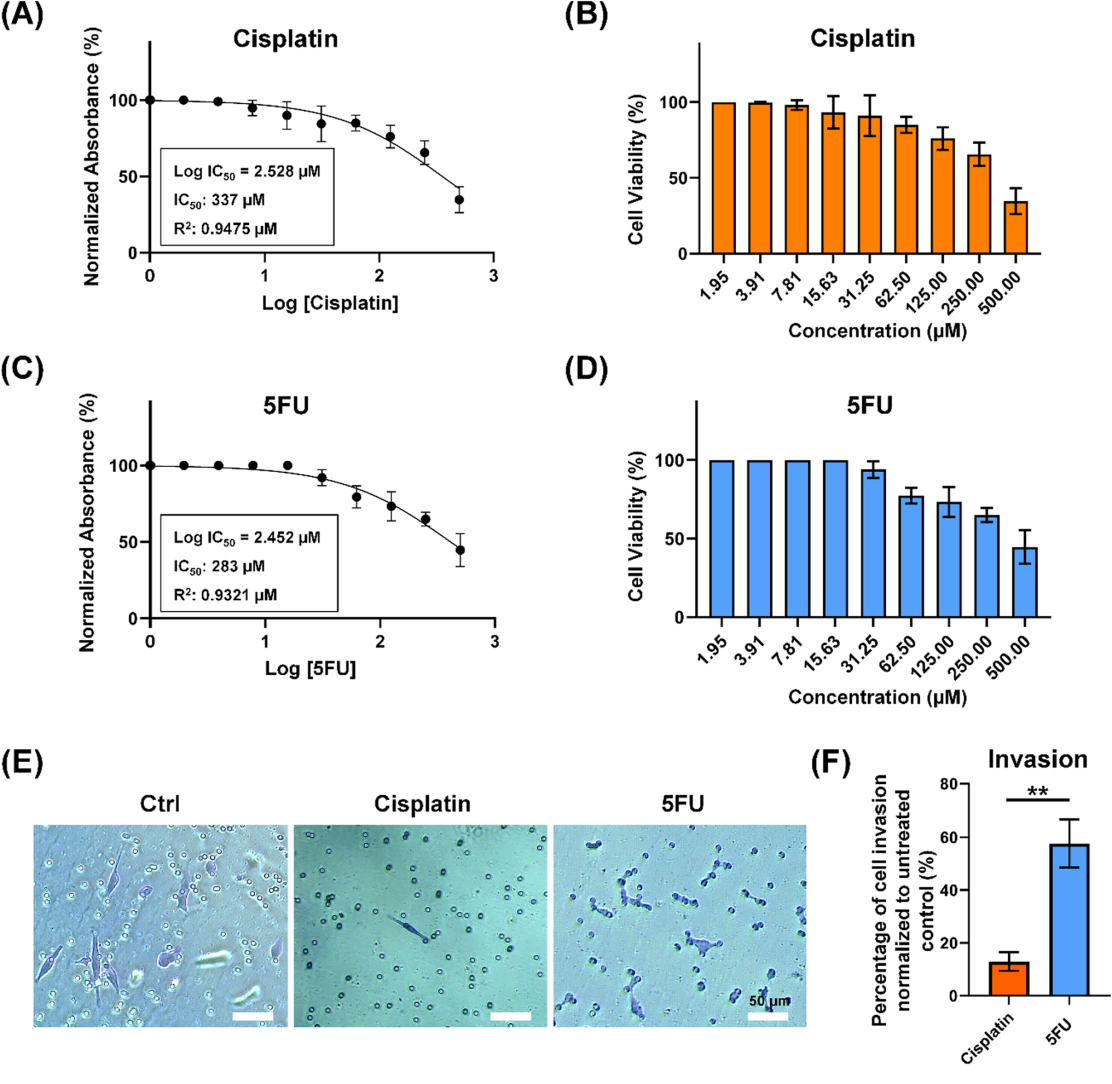
Cell invasion in MDA-MB-231 is inhibited by Cisplatin but not 5FU. **A** IC_50_ of Cisplatin after 24 h of drug treatment. **B** Average cell viability after 24-h Cisplatin treatment, *n* = 3 independent experiments. The brown bars represent the quantification of the average cell viability after 24 h of Cisplatin treatment. **C** IC_50_ of 5FU after 24 h of drug treatment. **D** Average cell viability after 24-h 5FU treatment, *n* = 3 independent experiments. The blue bars represent the quantification of the average cell viability after 24 h of 5FU treatment. **E** Transwell invasion assay of MDA-MB-231 (*n* = 3) for untreated cells (Ctrl) and drug-treated cells after 24 h. The phase-contrast image taken from the lower compartment of the Transwell shows migrating cells stained blue with a 0.5% crystal violet solution for visualization and quantification. **F** Quantification of Transwell invasion assay using MDA-MB-231 cells. Five randomly selected visual fields were analyzed per insert to determine the number of invading cells per Transwell. The percentage of invaded cells treated with cisplatin and 5FU was calculated by normalizing the count of drug-treated cells to that of their untreated vehicle control (MDA-MB-231 cells in 0.1% DMSO). Data are the mean ± SEM. Asterisks indicate statistically significant differences (Student *t* test, *p < 0.05, **p < 0.01) while n.s indicates statistically non-significant differences. Scale bar: 50 μm.

Prior to conducting the cell invasion assay, we first determined the IC_50_ values of MDA-MB-231 cell line using MTT assay for both Cisplatin and 5FU, to ensure that the selected drug concentrations did not induce cell death but rather inhibited the invasion properties of the MDA-MB-231. Figure 5A and B demonstrate that the IC_50_ for cisplatin is 337 µM and for 5FU is 283 µM, as depicted in Fig. 5C and D. We selected a concentration of 40 µM for both cisplatin and 5FU since cell viability remained above 90%, while observing changes in cell morphology and traction forces, indicating a potential reduction in invasion, particularly with Cisplatin.

After confirming that using 40 µM of both Cisplatin and 5FU did not induce cell death, we proceeded with the cell invasion assay as shown in Fig. 5E and F. To calculate cell invasion, we normalized the invaded cells to the untreated vehicle control condition (MDA-MB-231 in 0.1% DMSO), allowing us to accurately evaluate the relative change in invasion induced by different drug treatments. This enabled us to assess the efficacy of cisplatin and 5FU in modulating invasion capability, a critical aspect for understanding their potential therapeutic effects in cancer metastasis.

In Fig. 5E, the brightfield images revealed that 40 µM Cisplatin treatment resulted in a significant reduction in the invasiveness of MDA-MB-231 cells, as compared to 40 µM 5FU treatment. We observed a significantly lower percentage of MDA-MB-231 cell invasion in the Cisplatin-treated group when compared to the group treated with 5-FU, after normalization with the control. Specifically, approximately 12.94 ± 3.53% of cells invaded following Cisplatin treatment, while approximately 57.65 ± 9.09% of MDA-MB-231 cells invaded after 5FU treatment (Fig. 5F). Fluorouracil (5FU) is widely recognized as an anticancer drug known for disrupting nucleic acid synthesis, leading to cytotoxic effects and inhibiting proliferation without affecting invasion. However, our findings demonstrated that both Cisplatin and 5FU can inhibit the invasiveness of MDA-MB-231 to a certain extent. While a slight reduction in invasion was observed in the 5FU-treated cells compared to the control conditions, 5FU did not entirely halt the invasive behavior. This observation is supported by our findings in Fig. 5, where, at the same concentration (40 µM), Cisplatin appeared to exert more pronounced anti-invasive effects than 5FU, as evidenced by the significant reduction in MDA-MB-231 cells invading in the invasion assay. These invasion results suggested that Cisplatin effectively suppresses the invasive behavior of MDA-MB-231, subsequently contributing to a reduction in average cellular traction forces magnitude. Consequently, it also suggests that the decrease in cellular traction force is primarily attributed to the diminished invasiveness induced by Cisplatin treatment, rather than a reduction in proliferative activity caused by 5FU treatment. These results collectively confirm that cellular traction force has the potential to distinguish between antimetastatic and non-antimetastatic drugs for in vitro invasive cancer models.

## Discussion

Cancer metastasis is a crucial aspect of cancer progression and is responsible for the majority of cancer-related deaths [3, 23]. While most current preclinical models primarily focus on inhibiting cancer growth and proliferation as early-stage breast cancer studies, there are some research studies indicating that nearly one-third of patients progress to metastatic breast cancer even during these initial stages [6, 7].

Therefore, there is a growing interest in anti-metastatic agents. In this context, there exists an unmet need for in vitro models capable of predicting the anti-metastatic potential of drug candidates. However, the current preclinical drug discovery models, primarily oriented towards antiproliferative agents for primary tumors to inhibit cancer growth [24, 25], are inadequate for comprehensively modelling and studying cancer metastasis. This is mainly because the underlying mechanisms driving cancer metastasis and those governing tumor growth are very different. To initiate metastasis, cancer cells must undergo significant morphological changes from epithelial-like cells to mesenchymal morphology through a process known as epithelial-mesenchymal transition (EMT) [26–28]. This transformation enables cancer cells to generate sufficient cellular traction force through the actomyosin machinery, facilitating migration and invasion away from the primary tumor towards adjacent cells [29–31].

Cellular traction force is the force exerted by cells on their surrounding microenvironment to facilitate cell movement [32]. Although numerous studies have been conducted regarding traction forces in breast cancer cell lines, such as MCF-7 [33, 34] and MDA-MB-231 [12, 31], the utilization of cellular traction force measurements as a cellular marker for antimetastatic drug screening remains largely unexplored. Therefore, we aim to explore the potential of cellular traction force as a novel readout for drug screening in cancer metastasis.

To achieve our aim, it is important to comprehend the connection between the generation of cellular traction force and the potential for metastasis. Thus, we have opted to utilize a metastatic cancer model (MDA-MB 231) which is invasive, alongside a non-metastatic cancer model (MCF-7), which is non-invasive. This approach involves a comprehensive characterization encompassing cell morphology, involving factors like area, perimeter, cell aspect ratio, and circularity, as well as migratory capabilities through the utilization of transwell migration assays. Most importantly, we aim to perform the evaluation of the cellular traction forces exerted by both cancer cell lines.

Our observations revealed distinct patterns as the metastatic cell model, MDA-MB-231 cells, exhibited an elongated mesenchymal morphology, showcasing elevated migratory activity in the transwell migration assay. This translated to significantly higher traction force values for MDA-MB-231 compared to the more circular and epithelial-like morphology observed in MCF-7 cells, which displayed low migratory activity and correspondingly low traction force. These results suggest a correlation where increased traction force aligns with greater metastatic potential in a cancer model, while low traction force demonstrates reduced metastatic potential. Our finding aligns with existing literature, in which Kraning-Rush et al. demonstrated that metastatic lung, prostate, and breast cancer cells exert significantly higher cellular traction force corresponding with their non-metastatic counterparts [12].

Subsequently, we performed the drug screening process using the metastatic cell line MDA-MB-231 using two distinct compounds, which are the paradigmatic anti-metastatic drug Cisplatin, and the non-antimetastatic but anti-proliferative drug, 5FU. Interestingly, our findings demonstrated that Cisplatin, the antimetastatic agent, induced a reduction in cell size coupled with increased circularity. This was evidenced by the decrease in cellular area and the rise in circularity following Cisplatin treatment, observed in both MDA-MB-231 and MCF-7 cells.

In contrast, the application of 5FU, an anti-proliferation drug, resulted in diminished cell size, but it had a limited impact on cellular circularity. Particularly, cells in MDA-MB-231 and MCF-7 retained their elongated and circular morphologies, respectively, after the 5FU treatment. Therefore, it is evident that Cisplatin effectively restricts migratory activity in MDA-MB-231, whereas 5FU fails to exert the same effect. Taken all these together, our investigations unveiled that migratory activity correlates with cellular traction force, further underscoring its significance as an indicator.

We then further investigate if the cellular traction force could distinguish the effects of the antimetastatic drug Cisplatin from those of the non-antimetastatic drug 5FU. In this study, we do not conduct an invasion assay with MCF-7 cells due to their inherent non-invasive nature, which precludes invasion-like behavior. Interestingly, there was a significant reduction in cellular traction force observed in MDA-MB-231 cells after Cisplatin treatment, in contrast to the minimal impact seen in MCF-7 cells. This discrepancy strongly suggests that cellular traction force functions as a specific marker for the metastatic model, and not for the non-metastatic model.

Nevertheless, it is important to note that this current study serves as a proof-of-concept, demonstrating the potential of cellular traction force as an additional biophysical marker in the in vitro drug screening for cancer metastasis models, supplementing conventional migration and invasion studies. At this stage, focusing solely on one pair of positive and negative drugs with a single cancer model (MCF-7 and MDA-MB-231), limits our capacity to propose a definitive criterion for judging a new drug's efficacy. Therefore, our ongoing studies aim to establish stronger correlations between traction force alterations and observable changes in metastatic behavior across a larger array of drugs and diverse pairs of cancer models to confirm the feasibility of cellular traction force as an *in vitro* drug screening marker for antimetastatic drugs. Then, we can anticipate delineating robust correlations between traction force alterations and metastatic behavior, paving the way for proposing a criterion to judge the effectiveness of new drugs on metastasis.

## Conclusion

In conclusion, our study has successfully demonstrated the incorporation of cellular traction force measurement as an innovative and valuable marker for in vitro drug screening for cancer metastasis models in addition to the conventional migration and invasion study. This measurement offers the advantage of revealing not only the "effect" of cell movement but also the underlying "cause". As a result, our findings highlight the potential of cellular traction force as an indicator of metastatic potential, as increased metastatic capabilities correspond to increased cellular traction force. Cellular traction force also serves as a distinct marker for drug screening, specifically tailored to the metastatic model (MDA-MB-231), distinguishing it from the non-metastatic model (MCF-7) to differentiate the anti-metastatic drug from the non-antimestatic drug.

## Data Availability

The datasets used and/or analyzed in this study are accessible from the corresponding author upon reasonable request.
